# Socio-occupational class, region of birth and maternal age: influence on time to detection of cryptorchidism (undescended testes): a Danish nationwide register study

**DOI:** 10.1186/1471-2490-14-23

**Published:** 2014-02-28

**Authors:** Karin Sørig Hougaard, Ann Dyreborg Larsen, Harald Hannerz, Anne-Marie Nybo Andersen, Kristian Tore Jørgensen, Gunnar Vase Toft, Jens Peter Bonde, Morten Søndergaard Jensen

**Affiliations:** 1 The National Research Centre for the Working Environment, Lersø Parkallé 105, DK-2100 Copenhagen, Denmark; 2 Department of Occupational Medicine, Aarhus University Hospital, Aarhus, Denmark; 3 Department of Public Health, University of Copenhagen, Copenhagen, Denmark; 4 Department of Occupational and Environmental Medicine, Bispebjerg Hospital, Copenhagen University Hospital, Copenhagen, Denmark; 5 Perinatal Epidemiology Research Unit, Department of Paediatrics, Aarhus University Hospital, Aarhus, Denmark

**Keywords:** Cryptorchidism, Fertility, Diagnosis, Orchiopexy, Geography, Register, Public health

## Abstract

**Background:**

Cryptorchidism (undescended testes) is associated with poor male fertility, but can be alleviated and fertility preserved to some degree by early detection and treatment. Here we assess the influence of socio-occupational class, geographical region, maternal age and birth cohort on time to detection and correction of cryptorchidism.

**Methods:**

All boys born in Denmark, 1981 to 1987 or 1988 to 1994, with a diagnosis of cryptorchidism were identified in nationwide registers. The boys were followed for a diagnosis until their 16th birthday. The age at first diagnosis was noted and used as proxy for time to detection of cryptorchidism. Parental employment in the calendar year preceding birth was grouped into one of five socio-occupational classes. Geographical region was defined by place of birth in one of 15 Danish counties. Detection rate ratios of cryptorchidism were analyzed as a function of parental socio-occupational group, county, maternal age and birth cohort by use of Poisson regression.

**Results:**

Some 6,059 boys in the early and 5,947 boys in the late cohort received a diagnosis of cryptorchidism. Time to detection was independent of parental socio-occupational group and maternal age but differed slightly between geographical regions. A similar pattern was obtained for surgical correction after a diagnosis. Age at diagnosis decreased by 2.7 years from the early to the late cohort.

**Conclusions:**

These results indicate that childhood socio-occupational inequality in detection and correction of cryptorchidism would play a negligible role in male infertility in a life course perspective. Geographical region may have exerted some influence, especially for the oldest cohort.

## Background

Maldevelopment of the male reproductive tract is a major determinant of poor fertility later in life. Congenital cryptorchidism (undescended testes) is one such condition. Early treatment may alleviate the adverse effects of cryptorchidism on fertility, and the earlier orchiopexy is performed, the less the effect on future semen quality [1,2]. Adverse influence on gonadal cell populations in cryptorchid boys has been observed already at 3–6 months of age [3], and based on current literature, it is recommended that cryptorchidism is treated between 6 and 12 months of age [4].

Different long-term outcomes of cryptorchidism may arise from genetic, biological and environmental determinants, interacting with social, cultural and economic factors [5]. The Danish health care system is tax-financed and accessible for all Danish citizens. Therefore economic factors related directly to contact and treatment within the health care system would not be expected to significantly influence time to detection. A tax-financed health care system does however not remove all barriers to detection of disease. Time to detection of cryptorchidism might therefore be hypothesized to vary with parental socioeconomic factors such as competing priorities and time restraints, lack of information and knowledge, communication barriers, medical mistrust, health professionals’ perception of patients relative to socioeconomic status, and age [5-7]. Children of parents with low socioeconomic status are less likely to participate in the free preventive child examinations that are offered at the general practitioner until the age of five years [8]. The overall incidence of hospital referrals for cryptorchidism increases after each examination [9] and it has been estimated that testis palpation is performed in 60%-90% of these visits [10]. Non-adherence to routine prophylactic visits in low social classes may be an important reason for a delayed diagnosis and treatment. Furthermore, Danish hospitals (with few exceptions) were owned by the counties, and the treatments as well as the criteria for treatment may vary between counties as may regional differences in distance to hospital care [11-13].

Delayed diagnosis and treatment of cryptorchidism may affect male fertility in a life-course perspective. The present study investigates whether social class, maternal age and geographical region influence time to detection and surgical treatment of cryptorchidism. The study was performed in two cohorts, i.e. boys born in 1981 to 1987 or in 1988 to 1994, to investigate potential changes in the influence of social class and geographical region between these two time periods.

## Methods

### Study population and outcome

The study population consisted of singleton boys diagnosed with cryptorchidism before their 16th birthday. The boys were born in Denmark (excluding Greenland) from 1981 to 1987 and 1988 to 1994, i.e., the population was divided into two cohorts. At least one of the parents had to be employed in the calendar year preceding the year of birth.

The boys were identified through the Danish Civil Registration System [14], by the unique 10-digit identification number assigned at birth (CPR-number). The number is used across all public services in Denmark and enables linkage through population based registries, ensuring almost complete follow-up [15]. Cryptorchidism diagnoses were obtained from the Danish National Patient Registry, with nationwide coverage of all inpatient clinic diagnoses since 1977, and from 1995 also diagnoses from outpatient hospital clinics [16].

The boys were considered to have received a diagnosis if they had a record in the Danish National Patient Registry before their 16th birthday of a principal diagnosis of cryptorchidism according to the International Classification of Diseases version 8 (ICD-8) codes 752.10-752.11 and 752.19 or ICD version 10 codes Q53, Q531, Q531A, Q532, Q532A, and Q539. All diagnoses were made by hospital physicians, primarily paediatricians, general surgeons, urologists and paediatric urologists [14,17]. In diagnosed boys, treatment was defined by completion of the surgical procedure of orchiopexy (fastening of the undescended testicle inside the scrotum) as indicated by the corresponding surgical codes (described in [14]). Orchiopexy indicates that the cryptorchidism is persistent, while a general diagnosis of cryptorchidism may include transient cases of undescended testes that subsequently descend spontaneously.

We followed all boys for a diagnosis or surgery until their 16th birthday, which ensured comparable follow-up between cohorts and ascertainment of almost all cases of cryptorchidism (>95% of all cases presenting until the age of 30, in the oldest cohort). The age at principal diagnosis or surgery was noted. Since the earliest possible time for detection of cryptorchidism is at birth, time of detection corresponds to the age at detection. Age at detection was therefore used as a proxy for time to detection (TTD).

Statistics Denmark administered the data linkage and regulated data access. The data aggregation and analysis were approved by Statistics Denmark, the National Board of Health and the Danish Data Protection Agency. In accordance with Guidelines for Good Epidemiological Practice [18], a protocol outlining the methods was approved by the authors before the actual analyses were initiated.

### Explanatory variables

#### Parental socio-occupational status at birth of the child

Each boy was linked to his parents using the CPR-number in the Fertility Database [19], which enabled identification of parental employment as the basis for socioeconomic position [20,21]. In Denmark, all persons are classified annually according to occupation by Statistics Denmark from 16 years of age onwards. Specifically, socioeconomic position was defined by the NYSTGR classification, a specialised version of the Danish version the International Standard Classification of Occupations (ISCO) 1968 version, used from 1980 to 1995 by Statistics Denmark [22,23]. Socioeconomic position was defined the year before birth of the child, to avoid change in maternal occupational status due to maternity leave and change of occupational status, e.g. to become an (unemployed) house wife. The groups were defined and ranked as: white collar group 1 (senior salaried staff including managers etc. with more than 20 employees), white collar group 2 (leading salaried positions requiring higher academic education, i.e. generally more than 4 years beyond high school), white collar group 3 (other salaried staff, e.g. office and service workers, nurses, laboratory technicians, pre-school teachers), skilled workers, and unskilled workers. Since both maternal and paternal occupational levels contribute to the socioeconomic environment of the family, one common variable was constructed, using the highest occupational level from either the father or the mother as default [21]. In cases where the father could not be identified, maternal social class was used. Self-employed and individuals outside the workforce constitute two very heterogeneous groups that cannot readily be ranked with respect to socioeconomic position; the latter contains students, unemployed with various skilled background, house wives, and recipients of social security. We therefore included only children with at least one employed parent in the study population. If one parent was self-employed or outside the workforce, socioeconomic status of the employed (not self-employed) parent was used. Accordingly, children for which both parents were self-employed, outside the workforce, or self-employed combined with assisting spouse, were excluded from the study population. In total 730 out of 12,006 boys with cryptorchidism were excluded due to the above criteria. Table 1 presents the social status of the father and mother of the excluded boys.

**Table 1:** Exclusion of boys with cryptorchidism when none of the parents were registered as employee

	**Mother**			
**Father**	**Missing**	**Not economically active**	**Self employed**	**Total**
Missing	55	61	3	119
Not economically active	15	298	6	319
Self employed	4	116	172	292
Total	74	475	181	730

#### Geographical region at birth

TTD was assessed by region of birth, i.e. one of 15 Danish counties in existence between 1970 and 2006: Copenhagen, Frederiksborg, Roskilde, Western Zealand, Storstrøm, Bornholm, Funen, Southern Jutland, Ribe, Vejle, Ringkøbing, Aarhus, Viborg, Northern Jutland and the combined municipalities of Copenhagen and Frederiksberg. In essence, the region of birth corresponded to maternal region of living at the time of parturition. This variable was available for more than 99% of all children.

#### Maternal age at birth of the child

TTD was assessed by maternal age, divided into four categories (<25, 25–29, 30–34, ≥35 years).

### Statistical analysis

As stated, the age at principal diagnosis or surgery was used as a proxy for TTD. The detection rate of cryptorchidism (number of detected cases per person-year of in the cohorts of cryptorchid boys) as a function of socio-occupational group, county and maternal age was analysed by use of Poisson regression. Detection Rate Ratios (DRR) were estimated with unskilled manual workers as reference in analysis of socio-occupational status, and Copenhagen County as reference for geographical region. Maternal age at delivery was treated as a categorical variable with less than 25 years of age as reference. The DRR is a measure of relative difference that compares the detection rate of cryptorchidism in the group of interest with the detection rate in the reference group. The analysis was controlled for age of the child (in 1 year classes). Proportional hazards were assumed. Brothers were treated as repeated measurements. A first order autoregressive correlation structure AR(1) was assumed. The DRR estimates were stratified by birth cohort (1981–1987 and 1988–1994). Two-way interaction tests were performed to test whether effects of socio-occupational group, region, and maternal age at birth were independent of birth cohort. Follow-up ended at the time of diagnosis or surgical correction of cryptorchidism. The same statistical procedure was performed for cases with the surgical procedure of orchiopexy. Median TTD was estimated among the boys, by social group and region of birth.

The Poisson regression was performed by use of the GENMOD procedure in the statistical software package SAS version 9.2. The empiric standard error estimates was used. The significance level was set to 0.05.

## Results

In the early and the later cohorts, 6,059 and 5,947 boys, respectively, received a diagnosis of cryptorchidism. The mother’s personal identification number was missing for 19 of the boys and 730 boys were excluded due to parental occupational status, as described. The remaining 11,276 boys were included in the analysis. DRRs with 95% confidence interval (95% CI) are presented in Table 2.

**Table 2:** Detection rate ratios and median time to detection of cryptorchidism among cryptorchidism cases born in Denmark 1981–1994, by socio-occupational group, birth region and maternal age

	**1981-1987**				**1988-1994**			
	**N**	**TTD (years)**	**DRR**	**95% CI**	**N**	**TTD (years)**	**DRR**	**95% CI**
**Socio-occupational group**								
White collar 1	641	8.51	0.97	0.88 - 1.08	757	5.33	0.96	0.87 - 1.07
White collar 2	1112	8.20	1.00	0.92 - 1.08	1083	5.51	0.98	0.90 - 1.07
White collar 3	2203	8.27	1.02	0.95 - 1.09	1842	5.75	0.97	0.90 - 1.04
Skilled manual workers	658	7.92	1.11	1.01 - 1.21	617	5.59	1.00	0.91 - 1.11
Unskilled manual workers	1147	8.37	1.00		1216	5.45	1.00	
**County**								
Copenhagen (County)	665	7.71	1.00		725	5.43	1.00	
Copenhagen and Frederiksberg municipalities	411	8.03	0.98	0.86 - 1.11	480	5.48	0.95	0.84 - 1.07
Frederiksborg	328	8.89	0.76	0.67 - 0.86	352	5.76	0.95	0.84 - 1.07
Roskilde	220	8.04	0.91	0.78 - 1.07	189	6.45	0.83	0.72 - 0.96
Western Zealand	258	9.63	0.69	0.61 - 0.79	268	6.18	0.90	0.79 - 1.03
Storstrøm	263	8.39	0.75	0.65 - 0.87	286	5.31	1.04	0.90 - 1.21
Bornholm	48	9.58	0.65	0.49 - 0.85	27	5.63	0.90	0.65 - 1.25
Funen	557	7.57	0.92	0.82 - 1.04	546	6.07	0.90	0.81 - 1.00
Southern Jutland	307	8.86	0.76	0.66 - 0.86	266	5.24	1.17	1.02 - 1.34
Ribe	282	7.41	0.95	0.81 - 1.12	242	5.41	1.07	0.92 - 1.23
Vejle	381	8.15	0.86	0.76 - 0.98	335	5.88	0.90	0.79 - 1.03
Ringkøbing	338	8.81	0.77	0.68 - 0.88	319	5.91	0.89	0.78 - 1.02
Aarhus	853	8.06	0.90	0.81 - 1.00	764	5.14	1.07	0.96 - 1.20
Viborg	284	7.34	0.93	0.79 - 1.09	239	5.33	1.01	0.87 - 1.18
Northern Jutland	566	9.03	0.79	0.71 - 0.88	477	6.02	0.83	0.73 - 0.94
**Maternal age**								
<25	2033	8.24	1.00		1413	5.70	1.00	
25-29	2215	8.22	0.97	0.91 - 1.03	2248	5.46	1.04	0.97 - 1.12
30-34	1150	8.34	1.01	0.94 - 1.09	1391	5.64	0.96	0.89 - 1.04
>35	363	8.40	0.96	0.86 - 1.07	463	5.43	1.07	0.96 - 1.20

TTD: Time to detection (median value). DRR: Detection rate ratios.

**Figure 1:**
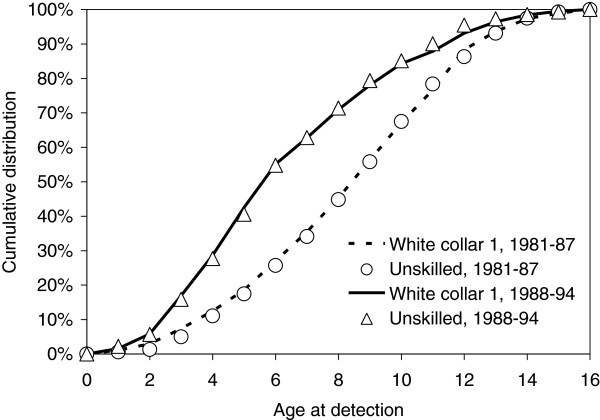
Cumulative distribution of age at detection of cryptorchidism (among boys with cryptorchidism detected before the 16th birthday), comparing the highest socio-occupational group (White collar 1) with the lowest socio-occupational group (Unskilled manual work), by birth cohort.

The effect of socio-occupational group was not statistically significant in any of the birth cohorts (P = 0.15 among the boys born in 1981 – 87; P = 0.85 among the boys born in 1988 – 94).The detection rates were highly dependent on birth cohort (P < 0.0001). The median TTD decreased from 8.37 years among the boys born 1981– 87 to 5.59 years among the boys born in 1988 – 94. The cumulative distribution of TDD for children in white collar group 1 and in unskilled manual workers by birth cohort is given in Figure 1.

Detection rates varied with county (P < 0.0001). The median TTD, by county, ranged from 7.3 to 9.63 years among the boys born 1981 – 87 and from 5.1 to 6.5 years among the boys born 1988 – 94. A statistically significant interaction between birth cohort and county (P < 0.0001) indicated that the effect of county was not constant and that some counties were slow in one period but faster in the other, and vice versa.

Maternal age at delivery was not statistically significantly associated with detection rate in any of the cohorts.

The analysis of orchiopexy was based on 4761 and 3705 boys from the early and later cohort, respectively. Results from this analysis did not challenge any of the statistical conclusions given above. These data are shown in Additional file 1: Table S1.

## Discussion and conclusions

The present study investigated the influence of socio-occupational class, geographical region and maternal age on time to detection of cryptorchidism. Rate of detection was independent of socio-occupational group in both birth cohorts. In fact, the cumulative distribution of TTD of cryptorchidism in children of employed parents in white collar group 1 almost exactly matched that in children of unskilled manual workers. There was no indication of a change in the influence of socio-occupational group between boys born 1981 to 1987 and 1988 to 1994. Detection rates did, however, vary between geographical regions. A similar pattern were obtained for surgical correction after a diagnosis.

These findings indicate that inequality in childhood socio-occupational status does not influence time to detection and correction of cryptorchidism in Denmark. The overall tendency of non-adherence to routine prophylactic visits by parents of low social classes thus seem to have only minor impact, if any, on the diagnosis and treatment of cryptorchidism [8,9]. It has, however, been shown, that although low income and education predict fewer contacts with specialist care, this may not be so for hospital referral. Actually, patients of low socioeconomic status may be referred relatively more frequently to hospital care than patients of high status [24,25]. This may somewhat compensate the less frequent use of preventive examinations in childhood at the general practitioners. Furthermore, detection of cryptorchidism does not entirely depend on visits at the general practitioner; A trained nurse specialised in child and family health care provides home visits following childbirth [11], and school-aged boys regularly attend the school nurse during the years of primary and lower secondary school in Denmark.

Some 7-20% of cryptorchidism cases cannot be detected at birth, and are said to be acquired [26,27]. Our study therefore implicitly assumes that potential differences in the incidence of acquired cryptorchidism have a negligible influence on the calculated rate ratios (including both congenital and acquired cryptorchidism). Knowledge about risk factors specific to acquired cryptorchidism is lacking but most cases of cryptorchidism, including acquired cases, are believed to occur due to prenatal causes [28]. We cannot rule out that the prevalence of acquired cryptorchidism differs by socio-occupational group, but the time-lag to detection of acquired cases may likely be similar to that of congenital cases; parents and general practitioners who act fast on congenital cryptorchidism may likely also act fast on acquired cryptorchidism. Taken together, we consider bias due to an uneven distribution of acquired cryptorchidism (if present) unlikely.

We observed shorter TTD in the more recent 1988–1994 cohort compared with the 1981–87 cohort. Median TTD decreased by more than 2.5 years (33%) between cohorts. This is in accordance with a similar trend in complete 1995–2009 birth cohorts [9]. Here mean age at diagnosis, when followed for 6 years, decreased from 3.3 years for boys born 1995 to 1997, to 2.9 years for children born 2001 to 2003 [9]. Collectively these data suggest a steadily decreasing median age at diagnosis and surgery throughout the period from 1981 to 2009. In addition, we observed a reduced variance in mean TTD between counties over time; in the 1981–87 cohort the difference between the fastest and slowest county was 2.5 years (mean TTD) and in the 1988–1994 cohort it was 1.3 years. This may indicate a more similar approach to cryptorchidism in all counties in the more recent cohort, as this difference decreased relatively (48%) more than the reduction in time to detection between cohorts. Among small children a difference in mean TTD of, e.g., one year may likely be clinically relevant [1,2]. Geographical region may therefore have exerted some influence on cryptorchid boys’ fertility later in life, especially in the oldest cohort.

The time to detection of cryptorchidism may be quite long. In the oldest cohort, 5% of the diagnosed cases remained to be found at the age of 16 years compared to the (full) follow-up of 30 years. In the most recent cohort, an even more complete follow-up would be expected to have occurred after 16 years, based on observations of the shift towards earlier diagnosis of cryptorchidism in recent years [9]. Follow-up was sufficient for almost complete case ascertainment. This allowed us to estimate the rates of diagnosis (time to detection) in different socio-occupational groups during the period of follow-up without bias from the total incidence [9,29]. Furthermore, the observed similar time to detection pattern in various socio-occupational groups allows for study of prevalence ratios of cryptorchidism across socio-occupational groups using a much shorter follow-up than in the present study.

The strengths of this study include diagnostic registry data based on standardised diagnostic reporting procedures and diagnosis by specialised hospital physicians [17]. The sample size was large and based on administrative registries of high quality with information on all Danish citizens, follow-up was almost complete, and a common coding system for socio-occupational class was used for all parents. The population based prospective birth cohort approach ensued that bias due to self-selection into the study was negligible, and e.g. language barriers were overcome. Boys, whose parents were both outside the workforce, students, self-employed or any combination hereof, were, however, not included. In total, 6% of the cryptorchid boys were excluded on these grounds. Our analyses do therefore not permit specific conclusions regarding boys from families where both parents were unemployed etc. High risk of delayed treatment in these non-participating groups seems unlikely, based on the almost exact match in age at detection of cryptorchidism between the most extreme socio-occupational groups in our study, but it cannot be ruled out completely.

The accuracy of the cryptorchidism diagnosis and surgery has been validated, based on review of a subset of medical records from 452 children born between 1995 and 2009 with a registry diagnosis of cryptorchidism, of which 249 underwent orchiopexy. The overall positive predictive value of a cryptorchidism diagnosis was 80%, and it was 99% for corrective surgery [17]. These children were born shortly after closure of our cohorts, and diagnoses were according to ICD-10. Otherwise this validation was performed on data with similar characteristics as in the present study.

In our oldest cohort, diagnosis and surgery were given on the same day for approximately half of the boys. In the younger cohort, less than 15% presented with diagnosis and orchiopexy on the same day. Until 1995, only inpatient diagnoses were available in the Danish National Patient Registry, where after also diagnoses for hospital outpatients became accessible. Inpatient admissions generally correspond to overnight hospital stays or daily hospital visits over an extended period, whereas outpatient admissions correspond to visits at hospital clinics on a less regular basis [29]. Most likely the diagnoses were given when the boys became inpatients due to surgery in the older cohort, whereas in the youngest cohort diagnoses were probably given already upon diagnostic consultation as outpatient. This may have contributed to the shift towards lower age at detection (diagnosis) in the younger cohort, but would not affect time to orchiopexy (which showed similar results).

We investigated effects of social status and geographical region in time to detection of cryptorchidism. Our findings indicate that childhood socio-occupational inequality in time to detection and correction of cryptorchidism would play a negligible role in male infertility in a life course perspective, which is reassuring from a public health perspective. Neither did we observe any association with maternal age. However, geographical region may have exerted some influence, especially for the oldest cohort.

## Competing interests

The authors declare that they have no competing interests.

## Authors’ contributions

KSH prepared the first protocol and manuscript together with HHA who conceived the idea and furthermore designed and performed the statistical analysis. KSH, HHA, ADL and MS contrived the coupling of data. Protocols and manuscripts were modified and adapted by ADL, MS, AMNA, KJ, GT and JPB. All authors read and approved the final manuscript.

## Pre-publication history

The pre-publication history for this paper can be accessed here:

http://www.biomedcentral.com/1471-2490/14/23/prepub

## Supplementary Material

Additional file 1: Table S1 Orchiopexy rate ratios and median time for orchiopexy among cryptorchidism cases born in Denmark 1981–1994, by socio-occupational group, birth region and maternal age. Click here for file
