# Supplementary material for: Socio-occupational class, region of birth and maternal age: influence on time to detection of cryptorchidism (undescended testes): a Danish nationwide register study
Source: BMC Urol. 2014 Feb 28;14:23. doi: 10.1186/1471-2490-14-23 (PMC4016268; doi:10.1186/1471-2490-14-23)
Supplement: Additional file 1: Table S1 — Orchiopexy rate ratios and median time for orchiopexy among cryptorchidism cases born in Denmark 1981–1994, by socio-occupational group, birth region and maternal age. [file 1471-2490-14-23-S1.pdf]

Table A1: Orchiopexy rate ratios (ORR) and median time to orchiopexy (TTO) among cryptorchidism cases born in Denmark 1981-1994, by socio-occupational group, birth region and maternal age

	1981 ï 1987				1988 - 1994			
	N	Median TTO	ORR	95% CI	N	Median TTO	ORR	95% CI
**Socio-occupational group**								
White collar 1	457	8.33	1.07	0.94 - 1.21	467	6.63	0.93	0.82 - 1.05
White collar 2	841	8.30	0.99	0.90 - 1.09	667	6.82	0.94	0.84 - 1.05
White collar 3	1681	8.50	1.02	0.94 - 1.11	1142	6.96	0.93	0.85 - 1.02
Skilled manual workers	522	8.10	1.09	0.98 - 1.21	390	6.57	0.98	0.87 - 1.12
Unskilled manual workers	875	8.43	1.00		722	6.68	1.00	
**County**								
Copenhagen (County)	410	8.36	1.00		385	6.63	1.00	
Copenhagen and Frederiksberg municipalities	303	8.24	1.08	0.93 - 1.25	278	6.39	0.98	0.83 - 1.14
Frederiksborg	238	8.88	0.94	0.81 - 1.09	211	6.80	1.02	0.87 - 1.19
Roskilde	141	8.88	0.84	0.70 - 1.00	110	7.83	0.78	0.63 - 0.95
Western Zealand	187	9.89	0.75	0.64 - 0.87	138	6.96	0.88	0.73 - 1.06
Storstrøm	178	8.23	0.91	0.76 - 1.09	146	7.12	0.94	0.78 - 1.12
Bornholm	41	8.70	0.84	0.59 - 1.19	22	6.53	0.98	0.66 - 1.47
Funen	451	7.64	1.16	1.02 - 1.32	360	7.23	0.91	0.79 - 1.05
Southern Jutland	249	8.73	0.87	0.75 - 1.01	173	5.77	1.16	0.97 - 1.39
Ribe	238	7.18	1.18	0.98 - 1.41	151	5.58	1.21	0.99 - 1.48
Vejle	330	8.33	0.97	0.84 - 1.12	250	6.16	1.03	0.87 - 1.22
Ringkøbing	264	8.75	0.90	0.78 - 1.05	217	6.90	0.98	0.83 - 1.16
Aarhus	674	7.94	1.04	0.92 - 1.18	474	6.71	1.00	0.87 - 1.16
Viborg	216	7.54	1.04	0.87 - 1.25	152	6.69	0.88	0.72 - 1.07
Northern Jutland	456	9.35	0.81	0.71 - 0.92	321	7.50	0.82	0.71 - 0.96
**Maternal age**								
<25	1539	8.48	1.00		840	6.80	1.00	
25-29	1700	8.26	0.99	0.93 - 1.07	1400	6.67	1.04	0.95 - 1.13
30-34	868	8.44	1.02	0.94 - 1.11	870	7.11	0.95	0.86 - 1.05
>35	269	8.44	0.96	0.84 - 1.09	278	6.64	1.14	1.00 - 1.30

TTO: Time to orchiopexy (median value). ORR: Orchiopexy rate ratios.
